# The “Far-West” of *Anopheles gambiae* Molecular Forms

**DOI:** 10.1371/journal.pone.0016415

**Published:** 2011-02-15

**Authors:** Beniamino Caputo, Federica Santolamazza, José L. Vicente, Davis C. Nwakanma, Musa Jawara, Katinka Palsson, Thomas Jaenson, Bradley J. White, Emiliano Mancini, Vincenzo Petrarca, David J. Conway, Nora J. Besansky, João Pinto, Alessandra della Torre

**Affiliations:** 1 Istituto Pasteur-Fondazione Cenci-Bolognetti, Dipartimento di Sanità Pubblica e Malattie Infettive, Università di Roma “Sapienza”, Rome, Italy; 2 Centro de Malária e outras Doenças Tropicais, UEI Malária and UEI Entomologia Médica, Instituto de Higiene e Medicina Tropical, Universidade Nova de Lisboa, Lisbon, Portugal; 3 Medical Research Council Laboratories, Banjul, The Gambia; 4 Medical Entomology Unit, Department of Systematic Biology, Uppsala University, Uppsala, Sweden; 5 Eck Institute for Global Health, Department of Biological Sciences, University of Notre Dame, Notre Dame, Indiana, United States of America; 6 Istituto Pasteur-Fondazione Cenci-Bolognetti, Dipartimento di Biologia e Biotecnologie “Charles Darwin”, Università di Roma “Sapienza”, Rome, Italy; The University of Queensland, St. Lucia, Australia

## Abstract

The main Afrotropical malaria vector, *Anopheles gambiae* sensu stricto, is undergoing a process of sympatric ecological diversification leading to at least two incipient species (the M and S molecular forms) showing heterogeneous levels of divergence across the genome. The physically unlinked centromeric regions on all three chromosomes of these closely related taxa contain fixed nucleotide differences which have been found in nearly complete linkage disequilibrium in geographic areas of no or low M-S hybridization. Assays diagnostic for SNP and structural differences between M and S forms in the three centromeric regions were applied in samples from the western extreme of their range of sympatry, the only area where high frequencies of putative M/S hybrids have been reported. The results reveal a level of admixture not observed in the rest of the range. In particular, we found: i) heterozygous genotypes at each marker, although at frequencies lower than expected under panmixia; ii) virtually all possible genotypic combinations between markers on different chromosomes, although genetic association was nevertheless detected; iii) discordant M and S genotypes at two X-linked markers near the centromere, suggestive of introgression and inter-locus recombination. These results could be indicative either of a secondary contact zone between M and S, or of the maintenance of ancestral polymorphisms. This issue and the perspectives opened by these results in the study of the M and S incipient speciation process are discussed.

## Introduction

*Anopheles gambiae* sensu stricto (hereafter *A. gambiae*) is the major mosquito vector responsible for malaria transmission throughout Sub-Saharan Africa. This species is undergoing a process of sympatric ecological diversification and lineage splitting which makes it a model to study divergent selection and heterogeneous genomic divergence mechanisms [1], [2]. Two morphologically indistinguishable incipient species (provisionally named M and S molecular forms) have been described within *A. gambiae*, recognized by form-specific SNPs on the IGS and ITS regions of multicopy rDNA located on the X-chromosome [3], [4]. The S-form is distributed across sub-Saharan Africa and breeds mostly in association with rain-dependent pools and temporary puddles. M-form populations overlap with the S-form in West and Central Africa, but are apparently absent to the east of the Great Rift Valley. M-form shows a greater ability to exploit breeding sites that exist across seasons and are more closely associated with human activities, such as those created by irrigation, rice cultivation and urbanisation [5], [6], [7], [8]. This adaptation allows the M-form to breed throughout the year, thus causing a shift from seasonal to year-round malaria transmission. Importantly, genetic traits conferring resistance to insecticides commonly used against these vectors are differently distributed between the two forms[9]. Therefore, beyond its intrinsic interest, the ecological speciation process occurring within *A. gambiae* also has practical consequences for malaria transmission and vector control in Africa [7], [8], [10].

The evolutionary and ecological forces that generated divergence between M- and S-forms are not yet fully understood. Lehmann & Diabaté [6] suggest that selection by larval predation and inter-form competition drove divergence between temporary and permanent freshwater habitats, possibly explaining the ecological discontinuity of the molecular forms (e.g. rice fields *vs.* surrounding savannas). The same authors also suggest that quantitative differences in adult body size, reproductive output, and longevity may have contributed to differential adaptations to distinct niches. Costantini *et al*. [7] showed that ecological segregation between M and S forms in Burkina Faso is consistent with a niche expansion of the M-form into marginal habitats. Altogether, these observations lead to the hypothesis that M and S forms are the products of divergent selection acting on ecologically important traits allowing optimal exploitation of permanent *vs*. temporary habitats for larval breeding [7], [8], [10], [11]. Theory predicts that such ecological diversification should lead to selection for reproductive isolation. Indeed, different pre-mating reproductive mechanisms between the two forms are reported, such as complete or almost complete swarm segregation based on visual landscape markers, as observed in Mali and Burkina Faso, respectively [6], [12], [13], and mating-recognition via matching of male-female flight-tone harmonic frequencies [14]. Field studies in Mali have shown that strictly sympatric and synchronously breeding populations of M and S forms cross-mate at a rate of only ca. 1% [15]. M/S hybrids (as detected by a SNP on the IGS-rDNA X-linked region) are exceedingly rare in the interior of west Africa (52 M/S hybrids among ∼18,000 *A. gambiae* identified [5], [7]) and absent from west-central Africa (none among >12,000 specimens identified [8], [16], [17]). Contrary to these findings, putative M/S hybrids have been recorded at much higher rates in westernmost west Africa (up to 3% in Senegal, [18]; 7% in The Gambia, [19]; and >20% in Guinea Bissau, [20]). Notably, crossing experiments reveal no detectable intrinsic postzygotic reproductive isolation between M and S in F1 or backcross individuals raised in the laboratory [21].

Whole genome scans of M and S divergence using a gene-based microarray have been performed on samples from Central and West Africa [22], [23]. High differentiation was detected almost exclusively in pericentromeric regions. These data were initially interpreted in the context of a speciation-with-gene-flow model that assumed introgression and homogenization of genetic variation outside of centromeric “speciation islands” [22]. However, the (nearly) complete genetic association of fixed allelic differences at centromeric markers on all three independently assorting chromosomes sampled from West, Central and East African populations is consistent with an alternative hypothesis, in which realized gene flow between forms is rare or nil across most of their range [23]. Importantly, this “hybridization-without-gene-flow” hypothesis is supported by more recent analyses based on whole genome sequencing and genotyping of M and S from Mali, indicating that heterogeneous divergence is genome-wide [24], [25].

The availability of diagnostic markers from the differentiated centromeric regions on all three chromosomes, and the expectation of their genetic association in the absence of realized gene flow, provide useful tools to study *A. gambiae* population structure in its extreme western range, where unusually high frequencies of putative hybrids between M and S-forms are found. We genotyped one marker in each centromeric island of genetic divergence (plus the IGS-marker defining M and S forms) in *A. gambiae* samples from The Gambia and Guinea Bissau, with the aim to evaluate the degree of reproductive isolation between M and S in this area, and to assess whether these unusual populations represent a recent breakdown in the M and S speciation process or whether they have simply retained ancestral polymorphisms. Persistent (though incomplete) genetic association between the unlinked pericentromeric markers, as well as discordant patterns observed for the two X-linked markers, seem to support the first hypothesis, opening new perspectives in the understanding of the molecular form speciation process.

## Materials and Methods

### *Anopheles gambiae* samples, genotyping and sequencing

**Figure 1:**
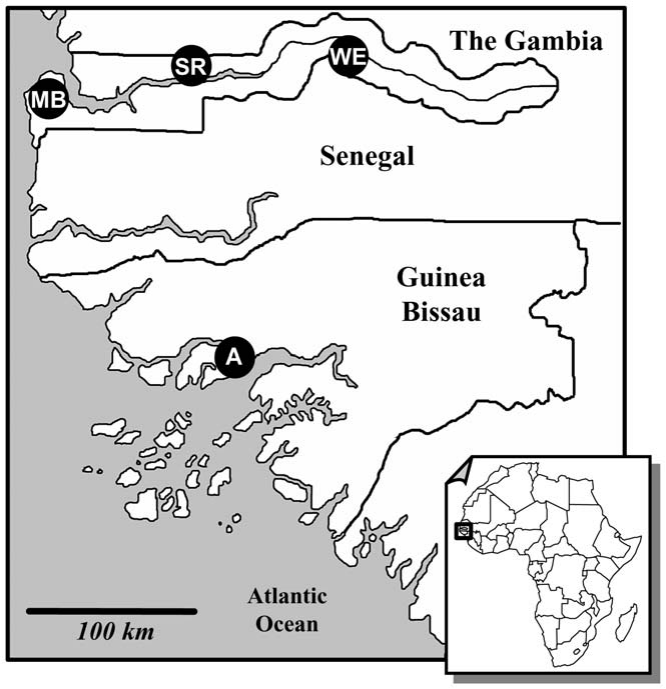
Map of the four collection sites in the Gambia and Guinea Bissau. [Footnote: MB = Mandina Ba; SR = Sare Samba Sowe; WE = Wellingara; A = Antula district of Bissau City].

We have analysed samples of indoor-resting *A. gambiae* females collected in three sites along the Gambia river (Mandina Ba, MB; Sare Samba Sowe, SR; Wellingara, WE, The Gambia) in 2006 [19] and in Antula district of Bissau City, Guinea Bissau, in 1995 (A-1995) and 1996 (A-1996) [20]. Each sample comprised specimens collected in a single catch (*i.e.* in the same site within 1-5 subsequent days), with the exception of samples from SR, which were collected in two catches in August and September. An additional sample analysed was collected in Antula district in 2007 (A-2007) (Figure 1).

Individual mosquito DNA was extracted from single legs or other parts of the carcasses not including the abdomen, to avoid the risk of contamination with DNA from sperm deposited in spermathecae. Samples were identified to species and molecular form by the PCR-RFLP approach recognising two form-specific SNPs in the IGS rDNA region on chromosome-X [26]. A subsample was also genotyped by the PCR-RFLP approach recognising an additional form-specific SNP in the IGS region, mapping only 109 bp apart from the former ones [27].

One molecular form-specific marker was analysed in each centromeric region, as follows: i) the M-form specific insertion of a SINE transposon in division 6 of the X-chromosome, detected by the PCR-approach developed by Santolamazza *et al.* [28] (hereafter termed SINE-X); ii) a SNP near the 2L-centromere in the fourth exon of AGAP004679 at position 2L:209 536 in the AgamP3 assembly (VectorBase; www.vectorbase.org), detected by a PCR-RFLP approach in which the M-form specific PCR-band is cleaved [23] (hereafter termed 2L); iii) a SNP near the 3L-centromere in the third exon of AGAP010313 at position 3L:296 923 in the AgamP3 assembly, detected by a PCR-RFLP approach in which the M-form specific PCR-band is cleaved [23] (hereafter termed 3L).

In addition, PCR amplicons from the IGS [29], 2L- and 3L-centromeric PCR assays [23] were sequenced from selected specimens, using ABI BigDye™ Terminator v2.0 chemistry and an ABI Prism 3700 DNA Analyser. Multiple alignments were performed using ClustalX [30]. Electropherograms were visually inspected for heterozygous SNPs. Sequences are available from the Authors upon request.

PCR and sequencing were carried out in Rome, Lisbon and Banjul. Selected samples were analysed in two different laboratories to validate the results.

### Statistical analyses

Estimates of inbreeding coefficient (F_IS_) were obtained according to Weir & Cockerham [31] using Genepop version 4.0 [32]. Departures from Hardy-Weinberg (HW) proportions were tested by exact probability tests implemented in ARLEQUIN v.3.5 with 1 million forecasted chain lengths and 10,000 dememorization steps [33]. The same software was used to perform tests of linkage disequilibrium (LD) between the 3 markers genotyped; both gametic phase unknown and known procedures were performed, despite the possible bias due to the location of the markers on physically unlinked chromosomes. For the first approach, we used a procedure that is based on a likelihood ratio test, where the likelihood of the sample evaluated under the hypothesis of no association (no LD) between loci is compared to the likelihood of the sample when the association is allowed [34]. In the second approach, we used the EM algorithm to estimate the maximum likelihood haplotype frequencies (see Arlequin manual 3.5) and calculated standard deviations through bootstrap followed by exact tests of LD based on the Markov chain approach (No. of steps in Markov chain = 100,000 and No. of dememorization steps = 1,000). Chi-square values were calculated using the VassarStat website for statistical computation (http://faculty.vassar.edu/lowry/VassarStats.html).

QSVanalyzer software - which was developed to facilitate the extraction of quantitative sequence variant (QSV) information from sequence electropherograms– was applied to estimate the relative proportions of the double peaks (*i.e*., **c**opy **n**umber **p**roportions: CNP) (http://dna.leeds.ac.uk/qsv; [35]) observed in electropherograms of IGS amplicon at positions 581 (M-form = T; S-form = C; [26], hereafter CNP^581^) and 690 (M-form = A; S-form = T; [27], hereafter CNP^690^) in sequences of the IGS locus from single *A. gambiae* specimens. The program analyses each trace and adjusts it in relation to the peak heights of upstream/downstream nucleotides, allowing rapid batch wise analysis of DNA sequence traces for estimation of the relative proportions of two QSVs at a given site. The CNP score (*i.e.*, the proportion between the heights of the C/T peaks at site 581 and 690) was calculated by dividing the S-form specific QSV by the sum of M- and S-form QSVs. Note that QSVanalyzer has never been applied before to analyse multicopy rDNA sequences. However, direct sequencing experiments revealed that the measurement of relative peak height of SNPs in the ITS regions represents an appropriate tool for studying hybridization in plants [36].

## Results

We genotyped one marker per centromeric region on each of the three independently assorting chromosomes (*i.e*., the M-form-specific SINE-X insertion on chromosome-X and two form-specific SNPs on chromosome-2 and -3) in *A. gambiae* populations from The Gambia [19] and Guinea Bissau [20].

### Chromosome-X

The results of the SINE-PCR analysis revealed the presence of M/S heterozygotes at this locus (hereafter, SINE-X^MS^), 1.7% in The Gambia (N = 304) and 22.9% in Guinea Bissau (N = 332). However, a significant SINE-X^MS^ deficit was detected in all samples analyzed (P<0.001), with the exception of Wellingara, where the SINE-X insertion was fixed (*i.e*., SINE-X^MM^) (Table 1).

**Table 1:** Frequencies of SINE-X genotypes in *Anopheles gambiae* adult female samples collected in The Gambia and in Guinea Bissau.

Country	Samples	SINE^MM^	SINE^MS^	SINE^SS^	N	F_is_	Exp. Heter.	P
The Gambia	MB	0.57	0.03	0.40	101	0.94	0.49	<0.001
	SR	0.54	0.01	0.44	153	0.97	0.50	<0.001
	WE	1.00	0.00	0.00	50	--	--	--
Guinea Bissau	A-1995	0.28	0.22	0.50	100	0.54	0.48	<0.001
	A-1996	0.43	0.25	0.32	79	0.49	0.49	<0.001
	A-2007	0.08	0.22	0.69	153	0.30	0.30	<0.001

*Footnotes:*

MB = Mandina Ba; SR = Sare Samba Sowe; WE = Wellingara; A = Antula district of Bissau City; N = sample size; F_is_ = inbreeding coefficient; Exp.Heter. = heterozygote frequencies as expected by Hardy Weinberg (HW) equilibrium; P = significance of deviation from HW equilibrium.

In contrast to what was observed elsewhere in Africa [28], results from SINE-X genotyping were not fully consistent with the identifications based on the IGS genotype that defines molecular forms. All specimens in which the IGS-based definition did not match the expected SINE-X genotype were confirmed at least twice by PCRs carried out in different labs. The final results verified mismatched genotypes in 4.7% (16/336) and 12% (40/332) of the Gambian and Guinean samples, respectively. The mismatches were of two types: (1) specimens defined by IGS as pure M (21.4%) and pure S (7.1%) showing a heterozygous SINE-X^MS^ genotype, or (2) specimens defined by IGS as M/S hybrids showing a homozygous SINE-X^MM^ (1.8%) or SINE-X^SS^ (67.9%) genotype. Only one specimen carried opposing homozygous genotypes (i.e. an IGS-based pure M-form specimen with a SINE-X^SS^ genotype); this was later shown to be characterised by IGS mixed array by the PCR-RFLP of a different IGS-SNP [27] and by direct sequencing of the IGS amplicon (see below).

To evaluate the occurrence of possible technical biases or, alternatively, the biological significance of the above-reported mismatched genotypes, we further PCR-RFLP genotyped a second form-specific SNP at position 690 in the IGS-locus [27] in a subsample of IGS/SINE discordant and concordant specimens. The results did not show a full agreement between the two PCR-RFLP approaches for IGS genotyping. Since the IGS region is known to be constituted by an array of tandem repeats subject to gene conversion and since the two IGS-SNPs co-segregate, we hypothesize that this lack of agreement is a product of a technical bias due to the fact that some individuals may contain an unequal number of tandem copies of the M- and S-form specific IGS-arrays (see Text S1 for details).

**Figure 2:**
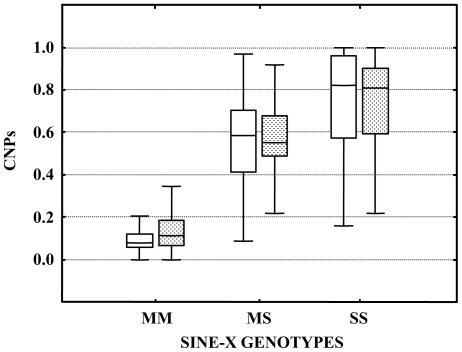
Box-plots of CNPs values at the IGS *Anopheles gambiae* molecular form-specific sites in SINE-X-genotypes. [Footnote: The vertical boxes in the plot include the data from the 1st to the 3rd quartile; the horizontal lines in the boxes are the median; the whiskers are drawn from the minimum to the maximum values. CNPs  =  Copy Number Proportions of IGS-SNPs at site 581 (white box) and 690 (grey box). Sample sizes: SINE-X^MM^ = 31, SINE-X^MS^ = 47, SINE-X^SS^ = 55].

To confirm the above hypothesis, we sequenced the IGS amplicon of 87 and 46 specimens in which the SINE-X and IGS genotypes did or did not match, respectively. High levels of C/T and A/T polymorphisms were observed in position 581 and 690 respectively, not only in heterozygous SINE-X^MS^ genotypes, but also in SINE-X^MM^ and SINE-X^SS^ homozygotes. The sequence electropherograms were further scored by QSV analyser [35] to quantify the proportion of sequences containing C *versus* T or A *versus* T, based on relative peak heights at position 581 (CNP^581^) and 690 (CNP^690^), respectively. M and S individuals from other geographical areas where hybridization rates are much lower show CNP scores ranging between 0-0.1 and 0.9-1, as expected if one allele is fixed (data not shown). Accordingly, F1 M/S hybrids are expected to show CNP scores around 0.5. However, CNP scores were highly variable and likely indicative of the co-presence of both the M- and S- IGS arrays not only in specimens with SINE-X^MS^ genotypes, but also in some specimens with SINE-X^MM^ or SINE-X^SS^ genotypes (Figure 2). The median CNP scores were statistically different among the three SINE-X genotypes (Kruskal-Wallis: CNP^581^: 2, 133 = 78.28, P<<0.001; CNP^690^: 2, 133 = 73.62, P<<0.001) and among pairs of SINE-X genotypes (Mann-Whitney test P values after Bonferroni correction <0.005). This suggests that a few SINE-X^MM^ and SINE-X^SS^ individuals may have “mixed” IGS arrays that are characterized by an unequal number of copies of M- and S-arrays.

### Chromosome-2 and -3

The results of the 2L-RFLP analysis revealed heterozygotes (hereafter 2L^MS^, *i.e.* individuals characterised by both the M and the S 2L-specific alleles) in all populations analyzed (113/298 specimens in The Gambia and 65/173 in Guinea Bissau). However, sequence analysis of the uncleaved 2L PCR products (N = 35) uncovered the presence of a second SNP in the restriction enzyme recognition sequence. This mutation, already described at very low frequency (<0.1%) in M-form populations from eastward geographic areas (Mali, Burkina Faso, Cameroon) by White *et al.* [23], is present in both forms in the study area, even in the homozygous state (freq = 29.2%). By altering the recognition sequence, this high frequency SNP acts as a kind of “null allele” that prevents straightforward interpretation of uncleaved PCR amplicons without extensive additional sequencing, a constraint that precluded full exploitation of this 2L marker in the complete set of samples.

The results of the 3L-RFLP analysis also revealed heterozygotes (hereafter 3L^MS^) in all populations analyzed (66/292 specimens in The Gambia and 92/322 in Guinea Bissau) (Table 2). Sequence analysis of the uncleaved 3L PCR products (in 28 3L^MS^, 44 3L^MM^ and 11 3L^SS^ individuals) ruled out second-site SNP mutations in the restriction enzyme recognition sequence. A significant deficit of 3L^MS^ heterozygotes was detected in all samples (P<0.001), with the exception of that collected in Guinea Bissau in 1995. In Wellingara (where all individuals were SINE-X^MM^ homozygotes), only 3L^MM^ and 3L^MS^ genotypes were detected, at a frequency of 52% and 48%, respectively.

**Table 2:** Frequencies of 3L genotypes in *Anopheles gambiae* adult female samples collected in The Gambia and in Guinea Bissau.

Country	Samples	3L^MM^	3L^MS^	3L^SS^	N	F_is_	Exp. Heter.	P
The Gambia	MB	0.54	0.15	0.31	97	0.68	0.48	<0.001
	SR	0.57	0.20	0.23	151	0.55	0.44	<0.001
	WE	0.52	0.48	0.00	44	−0.30	0.37	n.s
Guinea Bissau	A-1995	0.67	0.28	0.05	99	0.09	0.31	n.s
	A-1996	0.60	0.24	0.15	78	0.40	0.40	<0.001
	A-2007	0.37	0.31	0.32	145	0.38	0.50	<0.001

*Footnotes:*

MB = Mandina Ba; SR = Sare Samba Sowe; WE = Wellingara; A = Antula district of Bissau City; N = sample size; F_is_ = inbreeding coefficient; Exp.Heter. = heterozygote frequencies as expected by Hardy Weinberg (HW) equilibrium; P = significance of deviation from HW equilibrium; n.s. =  not significant.

### Association between chromosome-X, -2 and -3 centromeric regions

The complete association between X-, 2L- and 3L-centromeres observed by White *et al*. [23] in eastward geographic areas was not found in our populations (Table S1). Results from sequence analyses showed that most pair wise associations among X, 2L and 3L markers were present in the overall sample: 7 of 9 possible genotype associations between X and 2L, all possible associations between X and 3L, and 8 of 9 possible associations between 2L and 3L (Table S2).

Due to the high frequency of “null alleles” in both M and S at the 2L marker, only SINE-X and 3L markers were scored in the full set of samples (Table 3). The results show non-random associations of X and 3L genotypes in the whole Guinean sample (χ^2^ = 48.6; df = 4; P<0.0001) and in the Gambian one (χ^2^ = 179.4; df = 2; P<0.0001; NB: χ^2^ for the Gambian sample was calculated based on SINE-X^MM^ and SINE-X^SS^ genotypes only, due to the small size of SINE-X^MS^ sub-sample) (Table S3). Frequencies of putative “parental” genotypes (SINE-X^MM^-3L^MM^ and SINE-X^SS^-3L^SS^) were significantly higher than expected, while “assorted” genotypes (X^MM^-3L^SS^ and X^SS^-3L^MM^) were lower than expected. In particular, putative “parental” genotype frequencies were 34% in Guinea Bissau and 73% in The Gambia, while the corresponding frequencies of potential “F1” genotypes (SINE-X^MS^-3L^MS^) were 7.1% and 1%. Overall, the frequencies of “congruent” (X^MM^-3L^MM^, X^SS^-3L^SS^ and X^MS^-3L^MS^) genotypes were 41% in Guinea Bissau and 74% in The Gambia (χ^2^ = 67.1; df = 1; p<0.0001). Moreover, in Guinea Bissau the ratio of “parental” M SINE-X^MM^/3L^MM^ genotypes to “assorted” SINE-X^MM^/3L^MS^ or SINE-X^MM^/3L^SS^ genotypes was 6.3 to 1 (63/10), whereas the ratio of “parental” S SINE-X^SS^/3L^SS^ genotypes to the corresponding “assorted” genotypes was 0.3 to 1 (45/131) (χ^2^ = 75; df = 1; p<0.0001). In The Gambia, the above ratios were 4.8∶1 (150/31) and 1.5∶1 (64/43), respectively (χ^2^ = 17.5; df = 1; p<0.0001).

**Table 3:** Frequencies of diploid SINE-X/3L genotypes in *Anopheles gambiae* adult female samples collected in The Gambia and in Guinea Bissau.

			Parental genotypes		Assorted genotypes						
Countries	Samples	N	MM/MM	SS/SS	MS/MS	MM/MS	MM/SS	MS/MM	MS/SS	SS/MS	SS/MM
The Gambia	MB	97	0.515	0.309	0.010	0.052	0	0.010	0.000	0.093	0.010
	SR	151	0.510	0.225	0.007	0.033	0	0	0.007	0.159	0.060
	WE	44	0.523	0	0	0.477	0	0	0	0	0
	Tot	292	0.514	0.219	0.007	0.106	0	0.003	0.003	0.113	0.034
Guinea Bissau	A-1995	99	0.263	0.030	0.040	0.020	0	0.152	0.020	0.222	0.253
	A-1996	78	0.397	0.090	0.115	0.013	0.026	0.103	0.038	0.115	0.103
	A-2007	144	0.042	0.243	0.069	0.014	0.021	0.090	0.056	0.229	0.236
	Tot	321	0.196	0.140	0.072	0.016	0.016	0.112	0.040	0.199	0.209

*Footnotes:*

MB = Mandina Ba; SR = Sare Samba Sowe; WE = Wellingara; A = Antula district of Bissau City; N = sample size.

A significant association, as measured by the gametic phase unknown procedure, was observed between X and 3L loci in all samples (P<0.001), with the exception of that from Antula-2007. When measured by the gametic phase known procedure on inferred haplotypes (see Materials & Methods), the association was stronger in Gambian samples (MB: r^2^ = 0,67; SR: r^2^ = 0,48), than in Guinean ones (A-1995: r^2^ = 0,07; A-1996: r^2^ = 0,19). Results from inference analyses used to estimate the maximum likelihood of X/3L haplotypes, revealed frequencies of X^M^/3L^M^ and X^S^/3L^S^ haplotypes higher than expected under the hypothesis of linkage equilibrium (Table S4).

## Discussion

Previous studies have shown that in *A. gambiae* M and S molecular form populations from geographic areas of no or low (∼1%) inter-form crosses, such as and Cameroon, Burkina Faso and Mali, the physically unlinked centromeric regions of all three chromosomes contain fixed differences, which have been found in nearly complete linkage disequilibrium [22], [23], [37]. To date, only one marker in each centromeric region has been tested on a wide geographical scale: the SINE-X insertion unique to and fixed in the M-form [28] and two form-specific SNPs on chromosome-2L and -3L [23]. We analyzed these markers in populations from the western extreme of the *A. gambiae* range, the only area where high numbers of putative M/S hybrids have been reported thus far, and found much weaker genetic associations, but not panmixia between M and S. Most possible pairs of centromere associations were found, indicating that intrinsic genetic incompatible associations may not be considered responsible for the lack of finding of assorted genotypes along the *A. gambiae* range, as also shown in progenies from laboratory crosses and back-crosses (MW. Hahn, BJ. White, C. D. Muir, NJ. Besansky, unpublished).

The salient question is whether these results are best explained by secondary contact between M and S and partial breakdown of extrinsic mechanisms of isolation, or alternatively, by a less advanced speciation process characterized by a high degree of shared ancestral polymorphisms.

The high frequency of null alleles at the 2L marker precluded the scoring of markers on all three chromosomes in the full sample set, thus hindering one approach to addressing the above question, as F1 and backcross progeny could not be reliably distinguished. However, a more detailed consideration of patterns at the two X-linked markers (i.e. the IGS SNP which defines the M- and S-forms and the SINE-X insertion) from a molecular evolution perspective provides some support for the secondary contact hypothesis. First, the long-term maintenance of ancestral polymorphism would be unexpected near centromeres, given that centromere-proximal regions experience sharply reduced levels of recombination [38], [39]. Additionally, the rDNA locus consists of ∼1000 tandemly repeated genes, and concerted evolution is believed to be responsible for relatively rapid homogenization of sequence variation among genes in the array and between individuals in populations that are connected by sufficient gene flow. Thus, the presence of mixed (M+S) IGS arrays is expected to be transitory, and would be unlikely to persist at the high levels observed in this study, in an ancestral population. This is further supported by the virtual absence of mixed IGS in the rest of Africa. On the other hand, mixed IGS arrays in single individuals are a plausible outcome of recombination in M/S hybrids. With respect to SINE-X, no polymorphism has been observed at this locus during extensive surveys of M and S in other parts of Africa [28]: the insertion is fixed in all M form populations and absent in S. Because SINE elements cannot excise once inserted, the most parsimonious explanation for the absence of the SINE-X insertion in the S-form is that the element inserted into M after its divergence from S. Accordingly, the SINE-X polymorphism observed in both M and S in the study area is most likely the result of inter-form hybridization. Considering both loci jointly, the absence of individuals characterised by “opposite” IGS/SINE genotypes (i.e. M-form/SINE-X^SS^ or S-form/SINE-X^MM^) and the observation that SINE-X^MS^ individuals are more often characterized by M and S arrays at 50∶50 proportion (suggestive of F1 hybrids) than SINE-X^M^ and SINE-X^S^ ones (Fig. 2) further support the secondary contact hypothesis. Finally, the finding of SINE-X^MM^ and SINE-X^SS^ homozygous individuals characterized by mixed MS IGS-arrays suggests that crossing-over among the IGS-arrays allowed recombination within the X-centromere, despite the low rates reported for this region [38], [39].

The lower frequencies of putative parental genotypes and correspondingly higher hybrid frequencies observed in Guinea Bissau as opposed to The Gambia suggest that the former region could be the core (or be closer to the core) of the secondary contact zone. Intriguingly, we observed a lack of genetic association between chromosome-X and -3 centromeres only in the sample collected in Guinea Bissau in 2007. Genetic association between these centromeres was detected in earlier Guinean samples (collected in 1995 and 1996), and is very strong in Gambian samples. Although further investigation is required to confirm this speculation, these data seem to suggest both a geographic and a temporal trend, in which the lack of association observed in 2007 Guinean samples might be due to the weakening of inter-form reproductive barriers in the core of the secondary contact zone. Moreover, the data are consistent with the hypothesis that secondary contact could be the result of a (recent) invasion/colonization by S-form of the study area, where a long-established M-form population was present: i) the apparent higher variability in the ratio between M- and S-IGS arrays in SINE-X^SS^ compared to SINE-X^MM^ samples; ii) the greater match observed between SINE-X^MM^ and 3L^MM^ genotypes than among SINE-X^SS^ and 3L^SS^. Data on recent ecological changes in the region (e.g. changes in agricultural practices, urbanization) would be needed to further support this hypothesis.

The relative contribution of reduced pre- and post-mating barriers to inter-form gene flow in the study area is entirely unknown. In fact, this aspect of *A. gambiae* biology is still very poorly understood anywhere in the M and S range [6]. It may be possible that in the study area hybridization is not more frequent than elsewhere, but that negative selection is weaker against heterozygous genotypes and some genotype associations at the larval stage, resulting in greater survival to the adult stage (the stage most commonly sampled). Alternatively, reproductive barriers between the two molecular forms could be severely broken down, but negative selection could still be acting against heterozygous genotypes and favour some genotype associations. Field studies aimed at associating the centromere genotypes with biological parameters (e.g. assortative mating, inter-form insemination, larval development and survival, adult fitness) in the study area may shed light on the relative role of pre- and post-mating barriers in M and S speciation.

From the medical entomology perspective, the results - with particular reference to the finding of different copy number of M- and S-specific IGS-arrays in single individuals - highlight the weakness of the currently IGS-based definition of the two *A. gambiae* incipient species in areas where high frequencies of M/S IGS-patterns are found. This may strongly affect the interpretation of genetic analyses of populations such as those from Guinea Bissau and neighbouring areas. Therefore, until new markers will be developed, it would be advisable when working on *A. gambiae* populations from this region to simultaneously score all genetic markers available and to rely upon genetic associations maintained across the genome for their correct genotyping.

The first insights on the genetic constitution of the *A. gambiae* M and S populations from the western extreme of their range highlight the complexity and the variability of the biological and genetic differentiation of these incipient species in west-Africa. In fact, our current view of the incipient speciation process is widely affected by the limited geographic areas where most of the studies have been carried out (e.g. Mali, Burkina Faso, Cameroon), and the routine practice of identifying M and S mosquitoes based solely on a single IGS-based PCR-RFLP assay. More studies, using genome-wide approaches, are needed from other areas to have a more complete picture of this intriguing model of incipient speciation. The importance of this task goes beyond the goals of evolutionary biology, due to the medical importance of these species. It will be important, for instance, to evaluate and monitor the impact of the inter-form gene-flow we hypothesise is occurring in the study area on the dynamic of malaria transmission and the efficiency of vector control strategies.

## Supporting Information

Text S1 Genotyping of IGS -SNP^690^.
(DOC)

Table S1 Numbers (N) of 3-locus genotypes in the 35 *Anopheles gambiae* adult females whose 2L and 3L centromere genotypes were determined by direct sequencing.
(DOC)

Table S2 Pair wise associations of X, 2L and 3L centromeric regions, as determined by PCR detection of presence/absence of a SINE element insertion [28] and sequence analyses of form-specific SNPs in chromosome-2L and -3L centromeric regions [23] in *Anopheles gambiae* adult females from The Gambia and Guinea Bissau.
(DOC)

Table S3 Individuals with different SINE-X/3L genotypes observed and expected based on Hardy-Weinberg equilibrium in the *Anopheles gambiae* adult female samples collected in The Gambia and in Guinea Bissau.
(DOC)

Table S4 Frequencies and standard deviations of observed X and 3L centromeric region haplotypes inferred based on the frequencies of SINE-X and 3L genotypes in samples collected in The Gambia and in Guinea Bissau.
(DOC)
