# Supplementary material for: The “Far-West” of Anopheles gambiae Molecular Forms
Source: PLoS One. 2011 Feb 15;6(2):e16415. doi: 10.1371/journal.pone.0016415 (PMC3039643; doi:10.1371/journal.pone.0016415)
Supplement: Text S1 — Genotyping of IGS -SNP690. (DOC) [file pone.0016415.s001.doc]

**TEXT S1.**

To evaluate the occurrence of possible technical biases for the reported inconsistent results between M and S molecular form identifications based on the PCR-RFLP approach recognising a form specific SNP at position 581 in the IGS sequence (hereafter M^581^ and S^581^) [1] and the PCR-genotyping of the SINE-X locus [2], we further PCR-RFLP genotyped in a subsample of 63 discordant and 281 concordant specimens the IGS-SNP at position 690 (hereafter M^690^ and S^690^), co-segregating with the SNP at position 581 [3] .

All S^581^-form individuals showed a congruent S^690^ genotype (N=102), while M^581^- (N=143) and MS^581^-specimens (N=99) showed incongruent IGS^690^ genotypes in 26.6% and 19.2% of the analysed specimens, respectively. Inconsistently identified specimens were all either M^581^/MS^690^ (N=38) or MS^581^/S^690^ (N=19). Overall, 16.6% of the specimens showed an M/S heterozygote IGS-pattern only in one of the two IGS-SNPs analysed.

These inconsistencies are highly unexpected based on the extreme proximity of the two loci in the IGS region and on the results obtained in samples from other west-African regions [3]. We hypothesize that they may be due to the presence of an unequal number of copies of the M- and S-form specific IGS-arrays in single individuals, as already shown by Wilkins and colleagues in laboratory colonies [4]. In fact, it is plausible that this would introduce a technical bias due to the fact that the restriction enzymes used for the two PCR-RFLPs recognise either a S- [1] or an M-specific [3] restriction site, respectively. This would imply, for instance, that M^581^/MS^690^ individuals could be actually characterized by a overall number of copies of the M-IGS type higher than of the S-IGS type. After PCR amplification this difference could be increased, producing a strong M^581^ PCR-band and a faint S^581^ one that will not be visible on the agarose gel after the restriction step. This hypothesis is in full agreement with the absence of S^581^/MS^690^ and MS^581^/S^690^ genotypes.

The implication of these findings with respect to the reliability of currently applied methods for the identification of M and S forms along their range and the results of alignments of the entire IGS amplicon of all sequenced specimens will be discussed in a separate paper.

REFERENCES (also for Table S2)

1. Fanello C, Santolamazza F, della Torre A (2002) Simultaneous identification of species and molecular forms of the *Anopheles gambiae* complex by PCR-RFLP. Med Vet Entomol 16: 461-464.

2. Santolamazza F, Mancini E, Simard F, Qi Y, Tu Z, et al. (2008) Insertion polymorphisms of SINE200 retrotransposons within speciation islands of *Anopheles gambiae* molecular forms. Malar J 7: 163.

3. Santolamazza F, della Torre A, Caccone A (2004) Short report: A new polymerase chain reaction-restriction fragment length polymorphism method to identify *Anopheles arabiensis* from *An. gambiae* and its two molecular forms from degraded DNA templates or museum samples. Am J Trop Med Hyg 70: 604-606.

4. Wilkins EE, Howell PI, Benedict MQ (2007) X and Y chromosome inheritance and mixtures of rDNA intergenic spacer regions in *Anopheles gambiae*. Insect Mol Biol 16: 735-741.

5. White BJ, Cheng C, Simard F, Costantini C, Besansky NJ (2010) Genetic association of physically unlinked islands of genomic divergence in incipient species of *Anopheles gambiae*. Mol Ecol. 19: 925-939.
