# Supplementary material for: The “Far-West” of Anopheles gambiae Molecular Forms
Source: PLoS One. 2011 Feb 15;6(2):e16415. doi: 10.1371/journal.pone.0016415 (PMC3039643; doi:10.1371/journal.pone.0016415)
Supplement: Table S1 — Numbers (N) of 3-locus genotypes in the 35 Anopheles gambiae adult females whose 2L and 3L centromere genotypes were determined by direct sequencing. (DOC) [file pone.0016415.s002.doc]

**Table S1- Numbers (N) of 3-locus genotypes in the 35 *Anopheles gambiae* adult females whose 2L and 3L centromere genotypes were determined by direct sequencing.**

| 3-locus genotypes | |  |  |
| --- | --- | --- | --- |
| SINE-X | 3L | 2L | N |
| MM | MM | MM | 8 |
|  |  | MS | 3 |
|  |  | SS | 0 |
|  | MS | MM | 0 |
|  |  | MS | 1 |
|  |  | SS | 0 |
|  | SS | MM | 0 |
|  |  | MS | 0 |
|  |  | SS | 0 |
| MS | MM | MM | 0 |
|  |  | MS | 3 |
|  |  | SS | 0 |
|  | MS | MM | 2 |
|  |  | MS | 1 |
|  |  | SS | 0 |
|  | SS | MM | 0 |
|  |  | MS | 0 |
|  |  | SS | 0 |
| SS | MM | MM | 3 |
|  |  | MS | 2 |
|  |  | SS | 0 |
|  | MS | MM | 1 |
|  |  | MS | 2 |
|  |  | SS | 1 |
|  | SS | MM | 3 |
|  |  | MS | 4 |
|  |  | SS | 1 |
