# Supplementary material for: The “Far-West” of Anopheles gambiae Molecular Forms
Source: PLoS One. 2011 Feb 15;6(2):e16415. doi: 10.1371/journal.pone.0016415 (PMC3039643; doi:10.1371/journal.pone.0016415)
Supplement: Table S2 — Pair wise associations of X, 2L and 3L centromeric regions, as determined by PCR detection of presence/absence of a SINE element insertion [28] and sequence analyses of form-specific SNPs in chromosome-2L and -3L centromeric regions [23] in Anopheles gambiae adult females from The Gambia and Guinea Bissau. (DOC) [file pone.0016415.s003.doc]

**Table S2** – Pair wise associations of X, 2L and 3L centromeric regions, as determined by PCR detection of presence/absence of a SINE element insertion [2] and sequence analyses of form-specific SNPs in chromosome-2L and -3L centromeric regions [5] in *Anopheles gambiae* adult females from The Gambia and Guinea Bissau.

| Pair wise associations of centromeric markers | | | | | |  |  |  |
| --- | --- | --- | --- | --- | --- | --- | --- | --- |
| SINE-X/2L | | N | SINE-X/3L | | N | 3L/2L |  | N |
| MM | MM | 8 | MM | MM | 29 | MM | MM | 11 |
|  | MS | 4 |  | MS | 7 |  | MS | 8 |
|  | SS | 0 |  | SS | 1 |  | SS | 0 |
| MS | MM | 2 | MS | MM | 5 | MS | MM | 3 |
|  | MS | 4 |  | MS | 7 |  | MS | 4 |
|  | SS | 0 |  | SS | 1 |  | SS | 1 |
| SS | MM | 7 | SS | MM | 10 | SS | MM | 3 |
|  | MS | 8 |  | MS | 14 |  | MS | 4 |
|  | SS | 2 |  | SS | 9 |  | SS | 1 |
|  | Total | 35 |  | Total | 83 |  | Total | 35 |
