# Supplementary material for: The “Far-West” of Anopheles gambiae Molecular Forms
Source: PLoS One. 2011 Feb 15;6(2):e16415. doi: 10.1371/journal.pone.0016415 (PMC3039643; doi:10.1371/journal.pone.0016415)
Supplement: Table S3 — Individuals with different SINE-X/3L genotypes observed and expected based on Hardy-Weinberg equilibrium in the Anopheles gambiae adult female samples collected in The Gambia and in Guinea Bissau. (DOC) [file pone.0016415.s004.doc]

**Table S3 –** Numbers of individuals (N) with different SINE-X/3L genotypes observed (o), and expected (e) based on Hardy-Weinberg equilibrium, in the *Anopheles gambiae* adult female samples collected in The Gambia and in Guinea Bissau.

|  |  | The Gambia | |  |  | Guinea Bissau | |  |  |
| --- | --- | --- | --- | --- | --- | --- | --- | --- | --- |
|  |  | 3L^MM^ | 3L^SS^ | 3L^MS^ | Total | 3L^MM^ | 3L^SS^ | 3L^MS^ | Total |
| X^MM^ | o | 150 | 0 | 31 | 181 | 63 | 5 | 5 | 73 |
|  | e | 100.6 | 40.2 | 40.2 |  | 37.8 | 14.3 | 20.9 |  |
|  | (o-e)²/e | 24.3 | 40.2 | 2.1 | 66.6 | 16.9 | 6.1 | 12.1 | 35.1 |
| X^SS^ | o | 10 | 64 | 33 | 107 | 67 | 45 | 64 | 176 |
|  | e | 59.4 | 23.8 | 23.8 |  | 91.0 | 34.5 | 50.4 |  |
|  | (o-e)²/e | 41.1 | 68.0 | 3.6 | 112.7 | 6.3 | 3.2 | 3.6 | 13.1 |
| X^MS^ | o | 1 | 1 | 2 | 4 | 36 | 13 | 23 | 72 |
|  | e | - | - | - | - | 37.2 | 14.1 | 20.6 |  |
|  | (o-e)²/e | - | - | - | - | 0.0 | 0.1 | 0.3 | 0.4 |
| Total | o | 161 | 65 | 66 | 292 | 166 | 63 | 92 | 321 |
