# Supplementary material for: The “Far-West” of Anopheles gambiae Molecular Forms
Source: PLoS One. 2011 Feb 15;6(2):e16415. doi: 10.1371/journal.pone.0016415 (PMC3039643; doi:10.1371/journal.pone.0016415)
Supplement: Table S4 — Frequencies and standard deviations of observed X and 3L centromeric region haplotypes inferred based on the frequencies of SINE-X and 3L genotypes in samples collected in The Gambia and in Guinea Bissau. (DOC) [file pone.0016415.s005.doc]

**Table S4** – Frequencies and standard deviations of observed X and 3L centromeric region haplotypes inferred based on the frequencies of SINE-X and 3L genotypes in samples collected in The Gambia and in Guinea Bissau.

|  |  |  | SINE-X^M^/3L^M^ | | SINE-X^M^/3L^S^ | | SINE-X^S^/3L^M^ | | SINE-X^S^/3L^S^ | |
| --- | --- | --- | --- | --- | --- | --- | --- | --- | --- | --- |
| Countries | Samples | N | % Obs (sd) | %Exp | % Obs (sd) | %Exp | % Obs (sd) | %Exp | % Obs (sd) | %Exp |
| The Gambia | MB | 194 | 0.55 (0.04) | 0.35 | 0.03 (0.01) | 0.22 | 0.06 (0.02) | 0.26 | 0.36 (0.03) | 0.16 |
|  | SR | 302 | 0.53 (0.03) | 0.37 | 0.02 (0.01) | 0.18 | 0.14 (0.02) | 0.30 | 0.31 (0.03) | 0.15 |
| Guinea Bissau | A-1995 | 198 | 0.37 (0.04) | 0.31 | 0.02 (0.01) | 0.07 | 0.44 (0.04) | 0.49 | 0.17 (0.03) | 0.12 |
|  | A-1996 | 156 | 0.51 (0.04) | 0.41 | 0.02 (0.06) | 0.16 | 0.22 (0.03) | 0.32 | 0.22 (0.04) | 0.12 |
|  | A-2007 | 288 | 0.12 (0.02) | 0.10 | 0.07 (0.02) | 0.09 | 0.41 (0.03) | 0.43 | 0.41 (0.03) | 0.39 |

*Footnotes*:

MB=Mandina Ba; SR=Sare Samba Sowe; WE=Wellingara; A=Antula district of Bissau City; N= total numbers of alleles; %Obs=observed haplotype frequencies; sd=standard deviations; %Exp= frequencies of haplotypes expected under the hypothesis of a linkage equilibrium.
